# Familial silence surrounding HIV and non-disclosure of HIV status to older children and adolescents

**DOI:** 10.1080/09540121.2018.1434118

**Published:** 2018-02-04

**Authors:** Grace McHugh, Victoria Simms, Chido Dziva Chikwari, Hilda Mujuru, Kusum Nathoo, Prosper Chonzi, Shungu Munyati, Ethel Dauya, Tsitsi Bandason, Joanna Busza, Rashida A Ferrand

**Affiliations:** a Biomedical Research and Training Institute, Harare, Zimbabwe; b London School of Hygiene and Tropical Medicine, London, UK; c Department of Paediatrics, University of Zimbabwe, Harare, Zimbabwe; d Harare City Health, Harare, Zimbabwe

**Keywords:** HIV, children, adolescents, disclosure, caregivers, adolescents

## Abstract

Increasing numbers of children with HIV are surviving to adolescence and beyond, many of whom are orphaned. Disclosure of childrens' and adolescents' HIV status has been shown to improve adherence and retention in HIV treatment programmes. We investigated caregiving arrangements and intra-familial experience of HIV and its relationship to HIV disclosure to older children and adolescents. Children aged 6–15 years, newly diagnosed with HIV infection or previously diagnosed but not engaged in HIV care, were recruited from seven primary care clinics in Harare, Zimbabwe. Their caregivers responded to a nurse-led questionnaire. Family history of HIV, disclosure of HIV status to the child and reasons for non-disclosure were ascertained. The association between sociodemographics, caregiving, family HIV history and other characteristics and non-disclosure of HIV status to the child was determined using univariate and multivariate logistic regression. We recruited 385 participants, median age = 11 years (IQR: 9–13); 52% were female. Disclosure had occurred in 79% of children aged 11–15 years and 19% of children aged 6–10 years. Age under 11 years (adjusted OR [aOR] = 18.89, 95% confidence interval [CI] = 10.64–33.55; *p* < 0.001), being male [aOR]= 2.56, 95% CI = 1.49–4.54; *p *= 0.001, being unaware of the parents’ HIV status [aOR]= 32.42, 95% CI = 13.19–79.71; *p *< 0.001, and being newly diagnosed [aOR]= 2.52, 95% CI = 1.29–4.91; *p* = 0.007, were independently associated with non-disclosure. Disclosure outside of the family occurred infrequently and included friends of family (7%), school teacher (8%), school headmaster (4%) and church pastor (6%). High non-disclosure rates were present as well as a lack of discussion about HIV within the family. Disclosure outside of family was low reflecting difficulty in caregivers’ ability to discuss HIV with their child or surrounding community. HIV programmes need to support families in the disclosure process.

## Introduction

The global scale-up of antiretroviral therapy (ART) programmes has dramatically reduced mother-to-child transmission, reducing the number of incident infections in children as well as improving survival among those infected with HIV. This is shifting the paediatric HIV epidemic from one characterised by high early childhood mortality towards chronic infection in older children and adolescents (Ben-Farhat et al., 2017; Sohn & Hazra, 2013). It is estimated that in 2014, 1.8 million adolescents between 10 and 19 years old were living with HIV worldwide, the majority in sub-Saharan Africa (UNAIDS, 2016). Early ART programmes focused on provision of life-saving treatment, given the high early infant mortality. As children with HIV get older and particularly as they enter adolescence – a period of rapid cognitive, physical and psychological growth, their psychosocial needs evolve, and addressing these are key to children attaining successful treatment outcomes (Domek, 2006). One such vital component of HIV care for older children is disclosure of their HIV status to them. Knowledge of HIV status has been shown to be associated with improved adherence to ART and a higher rate of retention in care among children and adolescents (Arrivé et al., 2012). The World Health Organisation (WHO) recommends that partial disclosure begins from the age of 6–7 years (World Health Organsiation, 2011). Ideally, by adolescence, youth should know their status. Despite this, disclosure is often delayed, and studies have reported that the proportion of older children and adolescents with knowledge of their HIV status ranges from as low as 1.2% up to 75% at various stages of their HIV care, and appears to be lower in low- and middle- income countries (median 20.4%) (Pinzon-Iregui, Beck-Sague, & Malow, 2013).

The importance of the role of caregivers and families in the disclosure process cannot be overstated (Kidia et al., 2014; Mweemba et al., 2015). Caregivers are gatekeepers to children accessing HIV care, and influence children’s ability to take and adhere to treatment (Busza, Strode, Dauya, & Ferrand, 2016; Gross et al., 2015). We investigated caregiving arrangements and intra-familial experience of HIV and their association with non-disclosure of HIV status to older children and adolescents at time of their HIV diagnosis.

## Methods

This cross-sectional study was nested within a prospective cohort study aimed at investigating the outcomes of treatment among children aged 6–15 years from the time of diagnosis of HIV infection, in seven public sector primary healthcare clinics (PHCs) in southwest Harare, Zimbabwe (McHugh et al., 2016). Provider-initiated HIV testing and counselling (PITC), was offered to all children aged 6–15 years attending the study clinics. Children aged below 16 years attending unaccompanied by a caregiver were not eligible for HIV testing as per national guidelines. A caregiver was defined as an adult >18 years, responsible for the child’s daily care. Caregivers were informed of the HIV test results at the time of testing. Discussion of HIV test results with the child was undertaken only with caregiver consent. Children found to be HIV-positive were referred for care within the same clinic where HIV test was performed, and were offered enrolment into the study.

A detailed sociodemographic history including guardianship, orphanhood status, mode of HIV acquisition, enrolment and attendance at school was recorded at the initial assessment (performed within a week of HIV diagnosis using a nurse-administered questionnaire to child’s caregivers). In addition, the current and past caregiving arrangements of the child and history of HIV infection in parents and natural siblings was ascertained. Caregivers were asked whether the HIV status had been disclosed to the child and if disclosure had not occurred then reasons why disclosure did not happen were obtained using a pre-selected list. Caregivers could select more than one option and if the reason(s) for disclosure were not found in pre-selected list it was also documented. In addition, the child’s awareness of the parents’ and siblings’ HIV status, and whether the child’s HIV status had been disclosed to within and outside of family was also recorded.

Data were extracted from paper forms using optical mark recognition software (Cardiff TELEFORM Intelligent Character, Version 10.7), and analysed using STATA, version 12.1 (STATA Corporation, College Station, TX). The frequency of reasons for non-disclosure was calculated and the association between *a priori* defined variables and non-disclosure of HIV status to the child was determined using odds ratios, with all variables in univariate analysis included in a multivariate logistic regression model, to control for confounders. A *p*-value less than 0.05 was considered statistically significant.

Written informed consent to participate in the study was obtained from caregivers and age-appropriate assent obtained from participants ensuring that consenting process did not accidentally disclose HIV status to participant. Ethical approval for the study was obtained from the Medical Research Council of Zimbabwe, the Harare City Health Department Ethics Committee, the Biomedical Research and Training Institute Institutional Review Board and the London School of Hygiene and Tropical Medicine Ethics Committee.

## Results

We enrolled 385 children, of whom 52% were female and the median age at enrolment was 11 years, interquartile range (IQR) 8–13. The biological parent was the respondent to the questionnaire for 50% of participants. Almost all children (96%) were infected through mother-to-child transmission, and 23% of children had tested HIV positive in the past but had not engaged with care prior to enrolment into this study. The median age at enrolment in these previously tested children was 11 years and the median duration between initial HIV diagnosis and engagement with care through our study was 3.2 (IQR 0.14–13.0) years. School enrolment rates were high (91%), although nearly a quarter of participants had missed a week or more of school in the past three months, predominantly due to illness (Table 1).

**Table 1.** Participant socio-demographic characteristics at HIV diagnosis.

Variable	*N *= 385(%)
Age, y,median (IQR)	11 (8–13)
Sex,female	199 (52%)
*Orphanhood*	
Both parents alive	157 (41%)
Maternal Orphan^1^	150 (39%)
Paternal Orphan^2^	130 (34%)
Double Orphan	58 (15%)
*Current caregiver*	
Biological parent	220 (57%)
Nonparent Caregiver	165 (43%)
Aunt/Uncle	79 (48%)
Grandparent	61 (37%)
Sibling	17 (4%)
Other relative	4 (1%)
Institution	4 (1%)
Currently enrolled in school	351 (91%)
Missed ≥5 days school in past 3 months^3^	80 (23%)
-because of illness	61 (76%)
-financial reasons	10 (13%)
-other reason (relocation, death in family, teachers strike)	5 (5%)
-no reason given	4 (1%)
*HIV within the family*^4^*(alive or dead)*	
Both parents HIV+	140 (36%)
Mother HIV+	250 (65%)
Father HIV+	172 (45%)
One or more natural sibling HIV+^5^	51 (13%)

^1^Mother alive/dead/unknown by *n* = 3.

^2^Father alive/dead/unknown by *n *= 19.

^3^Data missing for *n *= 4.

^4^117 and 195 responders did not know mothers and fathers HIV status respectively.

^5^123 respondents did not know participants sibling HIV status.

Nearly 60% of children were single or double orphans, and 43% had a non-parent as the current primary caregiver (Table 1). The most common non-parental caregiver was an aunt or uncle (21%) followed by a grandparent (16%). Notably, 30% of children whose mother and/or father were alive lived with a non-parental caregiver. Caregiving arrangements were fluid and 57% of children had a change in caregiver since birth (43% having 1 change in caregiver, 13% 2 changes and 1% 3 changes). The main reason for change in caregiver was death of the child’s primary caregiver (*n* = 126, 57%).

The HIV status of 268 (70%) mothers and 190 (49%) fathers (either alive or deceased) was known to the caregiver. One hundred and forty (36%) caregivers reported that both the child’s parents were HIV+. Two hundred and four (53%) children had a parent who was on ART. Of the 250 (65%) mothers and 172 (45%) fathers whose HIV status was reported as positive by the caregiver, the children were aware of their mother’s HIV positive status in 41% of cases and father’s HIV positive status in 38% of cases. Three hundred and forty five children had 1 or more biological siblings; 51 (35 alive, 16 deceased) were known by the caregiver to be or to have been HIV-infected and 37 were currently receiving, or had received ART. Children were aware of their siblings’ HIV-positive status in 45% of cases.

Disclosure of their positive HIV status had been made to 202 (52%) children, with a higher proportion of 11–15 year olds than 6–10 year olds being told of their HIV diagnosis (79% vs. 19%) (Table 2). Disclosure to siblings and extended family members (i.e., a non-parent or non-sibling) had occurred in 31% and 63%, respectively (Table 2). Disclosure to individuals outside the family who could be sources of support (i.e., friends of family, school teacher, school headmasters and church pastors occurred infrequently.

**Table 2.** Reasons for non-disclosure (multiple reasons accepted).

	Total *n* = 385	6–10 years *n* = 185	11–15 years *n *= 200
HIV status not disclosed to child by caregiver	183 (48%)	146 (79%)	37 (19%)
Child too young	112 (62%)	100 (68%)	12 (32%)
Child doesn't understand	102 (56%)	86 (59%)	16 (43%)
May tell others his status	48 (26%)	39 (27%)	9 (24%)
May hurt the child to know his status	14 (8%)	5 (3%)	9 (24%)
Counsellor should disclose to child	10 (5%)	3 (2%)	7 (19%)
I don't know how to disclose to child	10 (5%)	6 (4%)	4 (11%)
Another relative should disclose to the child	8 (4%)	4 (3%)	4 (11%)
Child is not sick	2 (1%)	2 (1%)	0 (0%)
Child is too sick	2 (1%)	2 (1%)	0 (0%)

*n *= 11 no reason given.

The most common reasons for non-disclosure were the child’s young age, (*n *= 112 [62%]), caregiver’s concern about a child’s inability to understand the implications of an HIV diagnosis (*n *= 102 [56%]), and anxiety that the child would disclose the diagnosis to others (*n *= 48, [26%]). On univariate analysis, younger age, male gender, having both parents alive, no previous change in caregiver, having a parent as a caregiver, being unaware of parental HIV status and being newly diagnosed (as compared to having been diagnosed in a test previously) were associated with non-disclosure of HIV status to the participant (Table 3). On multivariate analysis, younger age, being male, being unaware of the parents’ HIV status and being newly diagnosed remained independently associated with non-disclosure (Table 3). Change of caregiver and being a non-orphan were associated with non-disclosure in univariate analysis but not in multivariate analysis, primarily due to confounding by orphanhood. Those in the older age group (10–15 years), but not in the younger age group (6–9 years), had higher odds of not knowing their status if newly diagnosed than previously diagnosed (Table 3).

**Table 3.** Risk factors for participants not knowing their status.

	Not disclosed to / Total (%)	Odds Ratio (95% C.I.)	*p*-value	Adjusted Odds Ratio (95% C.I.)	*p*-value
*Age*					
6–10y	149/185(79%)	16.5 (10.00–27.25)	<0.001	18.89 (10.64–33.55)	<0.001
≥11y	37/200(18%)				
*Sex*					
Female	81/199 (41%)	0.57 (0.38–0.85)	0.006	0.39 (0.22–0.67)	0.001
Male	102/186 (55%)				
*Orphanhood Status*					
Non orphan Maternal and/or	99/161(61%)	2.62 (1.73–3.98)	<0.001	1.50 (0.82–2.76)	0.19
Paternal Orphan	84/222 (38%)				
*Change of caregiver since birth*					
No previous caregiver change	90/162(55%)	1.75 (1.16–2.64)	0.008	1.00 (0.47–2.10)	0.99
≥1 change of caregiver	90/216 (42%)				
*Current Caregiver*					
Parent caregiver	114/220 (52%)	1.50 (1.0–2.25)	0.052	1.01 (0.47–2.16)	0.97
Non parent caregiver	69/165 (42%)				
*Disclosure of parents HIV status to child*					
Unaware of parents HIV status	171/260 (66%)	18.1 (9.47–34.58)	<0.001	32.42 (13.19–79.71)	<0.001
Awareness of parents HIV status	12/125 (10%)				
*Parent taking ART*					
Mother/Father not on ART	93/189 (49%)	1.14 (0.76–1.70)	0.52	1.64(0.90–2.98)	0.10
Mother/Father on ART	90/196 (46%)				
*WHO Stage*					
1/2	113/229 (49%)	1.20 (0.8–1.80)	0.39	1.28 (0.74–2.22)	0.37
3/4	70/156 (45%)				
*Schooling*					
Uninterrupted schooling in the last 3 months	132/271 (49%)	1.35 (0.82–2.24)	0.24	1.15 (0.58–2.26)	0.68
Interrupted Schooling in the last 3 months	33/80 (41%)				
*Time of diagnosis*					
Newly diagnosed	149/299(50%)	1.81 (1.08–3.02)	0.02	2.52 (1.29–4.91)	0.007
Previously diagnosed	28/79(35%)				

## Discussion

The main finding of our study is that caregivers of children living with HIV have difficulty in discussing HIV with their child resulting in high rates of non-disclosure to children after HIV diagnosis. Disclosure rates were low even to those children who had been diagnosed prior to testing in this study. WHO recommends that the disclosure process begins from age 6 years with age-appropriate discussions (World Health Organsiation, 2011). However, 29% of caregivers in our study were reluctant to broach the subject of HIV fearing the child was too young to understand. Females were more likely to be disclosed to than males, likely because girls are deemed to be more mature. Girls have sexual debut earlier than boys in this setting, and perhaps caregivers hoped to prevent onward transmission of HIV (Hallett et al., 2007; Pettifor, van der Straten, Dunbar, Shiboski, & Padian, 2004).

More than half of the children had one or more parents taking ART but this was not associated with disclosing to the child, and children who were not aware of their parents’ HIV status were also less likely to have been told about their own HIV-status. Previous studies have highlighted the fear that discussion of children’s HIV status will unmask parents HIV status and parents fear blame from their children for infecting them (Madiba, 2013; Mandalazi, Bandawe, & Umar, 2014; Waugh, 2003). Disclosure outside of the immediate family, including to school teachers who may be a potential source of support for the child, was also rare. This highlights the persisting culture of silence surrounding HIV. Caregivers may have wanted to protect the child from stigma or alternatively had concern that the child would disclose the diagnosis to others and subject the family to stigma (Kiwanuka, Mulogo, & Haberer, 2014; Mburu et al., 2014). However, children who learn their status recall that the initial feeling of shock and sadness at time of disclosure is relatively short-lived and believe they are in a stronger position to engage in ancillary support groups and be in control of their health after disclosure (Battles & Wiener, 2002; Mburu et al., 2014). Given that children rely predominantly on their caregivers for engagement with care and as the main source of information, their withholding of disclosure may affect the child’s capacity to adjust to the diagnosis when inevitably they do learn it. Delayed disclosure may promote self-stigmatisation in the child and in turn contribute to societal stigma.

Our study also highlights unstable living arrangements for children affected by HIV, with frequent change of caregivers, most often but not exclusively due to parental death. More than a fifth of caregivers were grandparents, many of whom are left to care for grandchildren following the death of their own children from HIV. Elderly guardians may have more socio-economic difficulties and lower HIV literacy leading to children in their care being unsupported (Skovdal, Campbell, Madanhire, Nyamukapa, & Gregson, 2011). In resource-constrained settings, children are left in care of extended family members while one or both parents leave home to seek employment due to economic hardships, resulting in changes to the traditional family structure (Manderson, Block, & Mkhwanazi, 2016). Caregivers are key to children both accessing and remaining in care and adhering to ART; changes in caregiving arrangements may interrupt children’s engagement with HIV care. Many children living with HIV have experienced the trauma and grief of family members dying including siblings and parents (Nyamukapa et al., 2008). Households can be fractured due to high rates of orphanhood and frequent change of caregiver (Manderson et al., 2016). Not discussing with a child their HIV status or HIV status of their parents/caregivers will potentially create further fracturing of relationships creating issues of trust between children and their guardians.

Caregivers play a central role in the process of disclosure, either by discussing the diagnosis with the child themselves or allowing health care providers to disclose. Even if disclosure is done by healthcare workers, this often amounts to only naming the diagnosis. Importantly, disclosure is not just a single event but an ongoing process – particularly for younger children – which must start at an age appropriate understanding and be escalated over time (Kidia et al., 2014; Kiwanuka et al., 2014; Mweemba et al., 2015; O’Malley et al., 2015). Naming the diagnosis will inevitably lead to questions and concerns from the child, and these often arise outside the clinical setting. Children often feel they receive minimal information about HIV from healthcare providers and their caregivers and the culture of silence surrounding HIV can leave them with many questions (Kidia et al., 2014; Mupambireyi, Bernays, Bwakura-Dangarembizi, & Cowan, 2014). Caregivers may lack knowledge on how to answer potential questions that may arise from their child once the child becomes aware of the diagnosis e.g., questions about death and about risk of transmission to others (Kiwanuka et al., 2014). Caregivers need to be equipped with the skills and support to be able to discuss HIV within their family unit openly and honestly in a way that is understandable to their children (Blasini et al., 2004). It would be assumed that this support would be provided by health care providers. However, health care workers may themselves lack the skills to discuss a child’s HIV diagnosis and may lack culturally appropriate methods (Blasini et al., 2004; Mokgatle & Madiba, 2015). There may also be a manpower shortage at clinics that can limit the time required to begin a disclosure discussion with both the caregiver and the child. In addition, they may be sanctioned by caregivers to not engage in discussion with the child. The process requires active engagement and ongoing collaboration between the caregiver and the healthcare provider. Given that the majority of children in the study were attending school – teachers can play an important role in health education and in particular HIV education.

A limitation of this study was that it was cross sectional, and therefore only provides a snapshot of disclosure following diagnosis. The cohort is being followed up to observe disclosure patterns over time. Study participants were by definition enrolled in care; it is likely that children who do not engage with HIV care services may be even less likely to know their HIV status.

It is essential that HIV care programmes are all encompassing- testing of HIV within families, open discussion of HIV status amongst family members and support of such family members through healthcare provider’s knowledge of HIV and counselling skills. As we move towards the 90-90-90 targets, we have a responsibility towards children and adolescents of today to inform them of their HIV status so as to empower them to be in control of its management and their own care (UNAIDS, 2014).
